# Metagenomic Virome Analysis of *Culex* Mosquitoes from Kenya and China

**DOI:** 10.3390/v10010030

**Published:** 2018-01-12

**Authors:** Evans Atoni, Yujuan Wang, Samuel Karungu, Cecilia Waruhiu, Ali Zohaib, Vincent Obanda, Bernard Agwanda, Morris Mutua, Han Xia, Zhiming Yuan

**Affiliations:** 1 Key Laboratory of Agricultural and Environmental Microbiology, Wuhan Institute of Virology, Chinese Academy of Sciences, Wuhan 430071, China; atonet@live.com (E.A.); wangyj1988@outlook.com (Y.W.); skarungu@yahoo.com (S.K.); hanxia@wh.iov.cn (H.X.); 2 Key Laboratory of Special Pathogens, Wuhan Institute of Virology, Chinese Academy of Sciences, Wuhan 430071, China; cecerumbs@gmail.com (C.W.); alizohaib7@hotmail.com (A.Z.); 3 University of Chinese Academy of Sciences, Beijing 100049, China; 4 Veterinary Services Department, Kenya Wildlife Service, P.O. Box 40241, Nairobi 00100, Kenya; vobanda@gmail.com; 5 Mammalogy Section, National Museum of Kenya, P.O. Box 40658, Nairobi 00100, Kenya; benrisky@gmail.com; 6 Entomology Section, National Museum of Kenya, P.O. Box 40658, Nairobi 00100, Kenya; mmutua17@yahoo.com

**Keywords:** *Culex quinquefasciatus*, *Culex tritaeniorhynchus*, virome, mosquito microbiome, metagenomics, emerging infectious diseases, insect-specific viruses

## Abstract

Many blood-feeding arthropods are known vectors of viruses that are a source of unprecedented global health concern. Mosquitoes are an integral part of these arthropod vectors. Advancements in next-generation sequencing and bioinformatics has expanded our knowledge on the richness of viruses harbored by arthropods. In the present study, we applied a metagenomic approach to determine the intercontinental virome diversity of *Culex quinquefasciatus* and *Culex tritaeniorhynchus* in Kwale, Kenya and provinces of Hubei and Yunnan in China. Our results showed that viromes from the three locations were strikingly diverse and comprised 30 virus families specific to vertebrates, invertebrates, plants, and protozoa as well as unclassified group of viruses. Though sampled at different times, both Kwale and Hubei mosquito viromes were dominated by vertebrate viruses, in contrast to the Yunnan mosquito virome, which was dominated by insect-specific viruses. However, each virome was unique in terms of virus proportions partly influenced by type of ingested meals (blood, nectar, plant sap, environment substrates). The dominant vertebrate virus family in the Kwale virome was *Papillomaviridae* (57%) while in Hubei it was *Herpesviridae* (30%) and the Yunnan virome was dominated by an unclassified viruses group (27%). Given that insect-specific viruses occur naturally in their hosts, they should be the basis for defining the viromes. Hence, the dominant insect-specific viruses in Kwale, Hubei, and Yunnan were *Baculoviridae*, *Nimaviridae* and *Iflaviridae*, respectively. Our study is preliminary but contributes to growing and much needed knowledge, as mosquito viromes could be manipulated to prevent and control pathogenic arboviruses.

## 1. Introduction

Global health security is increasingly threatened by emerging and re-emerging pathogens whose consequences to human and animal health are dire and devastating to economies. Most of these pathogens have their origin in diverse hosts, mainly wildlife, and are vectored by multiple blood-feeding arthropods. Part of the global health challenge is that some of these arthropods are widely distributed and highly efficient vectors for many pathogens. Specifically, the mosquito is one blood-feeding arthropod, known to feed on myriad hosts, and is an efficient vector for a multitude of virulent pathogens such as *Plasmodium* spp., filarial nematodes, Zika virus, dengue virus, Rift Valley fever virus and yellow fever virus. The diversity and richness of mosquito flora is only beginning to be understood following the application of next-generation sequencing (NGS) and vector-enabled metagenomics (VEM). These advanced molecular platforms have revealed that in addition to known pathogens, mosquitoes harbor a large proportion of organisms whose pathogenicity in vertebrate hosts is not yet known. Previous metagenomic studies have revealed that mosquitoes harbor a highly diverse microbiome composed of bacteria, protozoa, and viruses, of which some are specific to arthropods, protozoans, and plants [1,2,3,4]. The microbiome community of the mosquito is shaped by three broad factors; the vector itself, in vivo microorganisms in the mosquito body, and lastly the mosquito meal sources.

There is a developing specific interest to understand mosquito viromes, particularly in terms of virus diversity and composition. This arises from the fact that most mosquito-borne viruses bear significant threat to public and animal health, whereas a large proportion (<70% amino acid identity) of recovered viruses are of unknown pathogenicity [5,6]. Surveillance of animal and human viruses to determine the composition and proportion of circulating viruses in specific mosquitoes within a region is a necessary challenge because the outcome is fundamental and critical for formulating strategies on prediction, preparedness, prevention, and eventual control of any disease. Thus, mosquito virome studies have been proposed as a promising robust tool that could be applied in virus surveillance [1]. This is based on the fact that both DNA and RNA viruses can be detected from mosquito blood meal for up to 24 h [7], coupled by the reality that the diversity includes viruses not biologically vectored by the specific mosquito.

Besides viruses that can replicate in vertebrates, mosquito viromes seem to harbor a large proportion of viruses that naturally infect insects and can replicate only in insect cell lines in vitro. Even though these insect-specific viruses seem not to replicate in vertebrates [8], some of them are phylogenetically related to known pathogenic arboviruses and it is speculated that they may evolve to become novel pathogenic viruses in vertebrates [9]. Further, due to their close evolutionary association with known arboviruses, they are being hypothesized for potential biocontrol applications (modulation of transmission of arboviruses) or as a basis for new vaccine formulations [9,10,11,12]. Nevertheless, due to their abundance and diversity (including the virus families *Togaviridae*, *Flaviviridae*, *Rhabdoviridae*, *Bunyaviridae* and *Mesoniviridae*), they constitute a significant component of mosquito viromes. In addition, it seems that each virome is dominated by particular viruses, which hypothetically may be transient, but significant in distinguishing or describing diverse viromes. For example, the virome of *Culex* spp. in Zambezi, Mozambique was dominated (88.5%) by insect-specific viruses, of the *Iflaviridae* family [13]. In China, a previous study of *Culex tritaeniorhynchus* virome in Hubei province also showed a similarly high dominance (88%) of insect-specific viruses [4].

Increased frequency and speed of human travel and commercial trade has been implicated in the expansion of mosquito vectors and their pathogens from indigenous habitats to new areas. The well-known *Aedes albopictus* first made its incursion to South Africa through trade in old car tires [14] and has since been established in Africa. We hypothesized that because of the increased interaction between China and Kenya, there is high likelihood for exchange of both mosquito and viruses. For instance, Ochieng et al. (2013) isolated a flavivirus from Rabai town, coastal Kenya that had 77% nucleotide similarity to Chaoyang virus from Shenyang, China. Therefore, our objective was to determine viromes of *Culex* mosquitoes in specific sites in China and Kenya. The *Culex* mosquito is an efficient vector for several arboviruses such as West Nile virus, Rift Valley fever virus, and Japanese encephalitis, as well as parasitic bancroftian filariasis. African mosquito virome studies are in their nascent stage, with few exploratory investigations, though outcomes from such studies show remarkable diversity and composition [2,13]. Our study will provide important preliminary information on virus status in populations of *Culex* spp. in both Kenya and China.

## 2. Materials and Methods

### 2.1. Study Sampling Sites and Related Important Ecological Features

The study areas in Kenya were located in the Kwale and Kilifi counties, along the Indian Ocean coastline. The study areas in China were located in the Hubei province in south-central china and the Yunnan provinces in south-western China.

#### 2.1.1. Kwale County—Kenya

**Figure 1:**
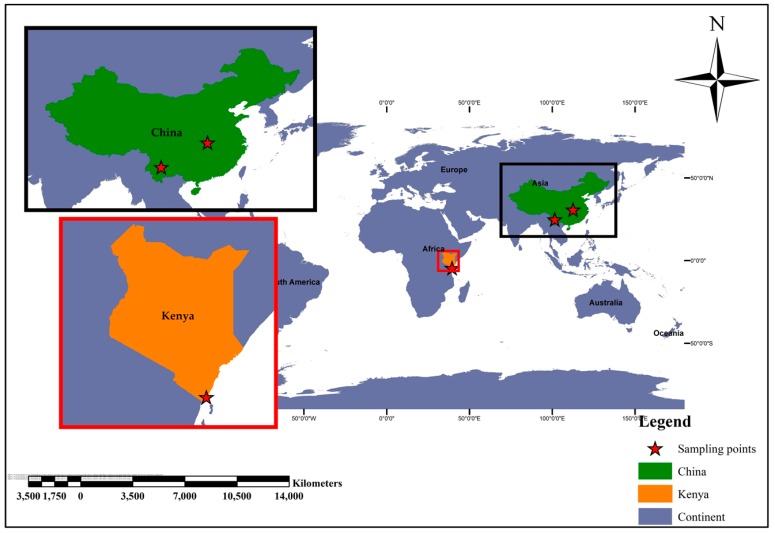
World geographical map outlining the geo-location of Kenya and China. Kenya is outlined in orange in the African continent while China is outlined in green in the Asian continent. The red stars represent sampling sites.

Kwale county is located on the south-eastern part of Kenya along the shores of the Indian ocean (Figure 1). It lies at 5 m (18 ft) above sea level. It has a tropical humid and temperate climate all year. The annual mean temperature is 26.3 °C (79.3 °F). In the months of January and February, the average temperature is 31 °C (88 °F) and an average precipitation of 22 mm (Source: Kenya Meteorological Department). The forest cover values for the three counties flagging the Indian ocean coastline are 5.44%, 7.67% and 33.9% for Kwale county, Kilifi country and Lamu county, respectively (Source: http://www.opendata.go.ke). The region has experienced several arbovirus outbreaks due to its high human population together with high inflow and outflow of visiting tourists. Also, the close vector/human/wildlife proximity might be a contributing factor to the emerging outbreaks in the region.

#### 2.1.2. Kilifi County—Kenya

Kilifi county lies along the coastal belt, in the south-eastern part of Kenya. The region receives an average annual rainfall of 900–1100 mm. The annual temperatures ranges from 21 °C to 30 °C in the coastal belt but rise to maximum of 34 °C in the hinterland (Source: http://www.kilifi.go.ke). There is a precipitation difference of 246 mm between the driest and wettest months. The annual temperature variation is 3.8 °C. Due to its proximity to the Indian Ocean, tourism and fishing are the main economic activities hence experiencing a high human traffic.

#### 2.1.3. Hubei Province—China

Hubei Province is located in south-central China (Figure 1). It has extreme weather conditions, (more so during the summer) and is humid all year round due to high number of water bodies in the region. It features a subtropical monsoon climate with an average annual temperature of about 15 °C (59 °F). The province has four distinct seasons with a burning hot summer (June, July and August) and a chilly winter (December, January and February). The hottest month, July, averages 27–30 °C and the coldest month, January, 1–5 °C. The average annual precipitation is between 800–1600 mm. In Hubei, summer is the wettest season with 300–700 mm in average precipitation, while winter is the driest season with average rainfall at 30–190 mm. The average temperature for the months of July to September is 30 °C (83 °F) (Source: http://en.hubei.gov.cn).

#### 2.1.4. Yunnan Province—China

Yunnan Province is located in the south-western part of China (Figure 1) and it has a characteristic feature of “spring-like” weather with very good forest cover. The region sits at an altitude of 1000 m above sea level with abundant heat and light, a spring-like climate. The annual average temperature is 17 °C. The average temperature for the months of July to Sept is 22 °C and average precipitation is 21 mm. Yunnan province in China serves as the main business trading route between countries in Southeast Asia and China and it shares a lengthy border with three countries: Myanmar, Laos and Vietnam. This region is seen as the main epicenter of emerging or re-emerging viral diseases due to its multi-ethnic society and poorly developed public health structures, as well as abundant and diverse wild animal resources and their illegal trading [15,16,17,18].

### 2.2. Sample Collection and Taxonomic Identification

Mosquito trappings for adult stages were set at randomly selected sites in Kwale and Kilifi county, on the Kenyan coast. The mosquitoes were trapped alive using carbon dioxide mosquito traps (Mosquito Magnet^®^ Woodstream Corp., Brampton, ON, Canada) from outdoor sites between dusk and dawn, in the months of January to February 2017. In China, adult mosquitoes were sampled from two provinces, namely the Hubei province (Coordinates: 30.8845° N, 112.5923° E) and Yunnan province (Coordinates: 24.97564° N, 101.4848° E) (Figure 1). In Hubei, sampling was done between the month of July and September of the year 2014 while in the Yunnan province it was done from July to September of the year 2015. In these two sites in china, electrical mosquito aspirators (Yalin, Zhejiang, China) were used in mosquito capture from homesteads and surrounding regions. In all the study sites, the mosquitoes were killed by dipping in liquid nitrogen. The killed mosquitoes were morphologically identified as described by Edwards (1941) and Hopkins (1952) [19,20]. The mosquitoes were grouped into pools of 50 and stored at −80 °C until further analysis.

### 2.3. Viral RNA Isolation, cDNA Library Preparation, Amplification and Sequencing

The mosquito pools were homogenized in 1 ml Roswell Park Memorial Institute medium (RPMI) and the homogenate filtered through a 0.22 μM polycarbonate filter membrane (Millipore, Billerica, MA, USA) to exclude bacteria and other debris. RNA was extracted from 140 μL of homogenate using the QIAamp viral RNA extraction kit according to manufacturer’s instructions (Qiagen, GmBH, Hilden, Germany). The RNA integrity was checked using the Agilent bioanalyzer 2100 (Agilent Tech, Santa Clara, CA, USA) and further purified using the RNA clean XP kit (Beckham Coulter Inc., Brea, CA, USA). Strand-specific libraries were prepared using the TruSeq^®^ Stranded Total RNA Sample Preparation kit (Illumina, San Diego, CA, USA) following the manufacturer’s instructions. Briefly, ribosomal RNA was removed from total RNA using Ribo-Zero rRNA removal beads. Following purification, the mRNA was fragmented into small pieces using divalent cations under 94 °C for 8 min. The cleaved RNA fragments were copied into first strand cDNA using reverse transcriptase and random primers. This was followed by second strand cDNA synthesis using DNA Polymerase I and RNase H. These cDNA fragments then went through an end repair process, the addition of a single “A” base, and then ligation of the adapters. The products were then purified and enriched with PCR to create the final cDNA library. Purified libraries were quantified by Qubit^®^ 2.0 Fluorometer (Life Technologies, Waltham, MA, USA) and validated by Agilent 2100 bioanalyzer (Agilent Technologies, Santa Clara, CA, USA) to confirm the insert size and calculate the mole concentration. Cluster was generated by cBot with the library diluted to 10 pM and then were sequenced on the Illumina HiSeq 2500 (Illumina, San Diego, CA, USA). The library construction and sequencing was performed at Shanghai Biotechnology Corporation.

### 2.4. Bioinformatics Analysis

Bioinformatics analysis was performed as described by Guillaume et al. [21] but with slight modifications. Briefly, library adapters were trimmed from the sequences in the fastq file and the reads below 50 bp were discarded. The host genome was removed by mapping to the *Culex* genomes with Bowtie2 [22]. The sequence reads that did not match the host genome were retained and subsequently used for de novo assembly in the “Trinity” tool [23]. These de novo assembled contigs were then subjected to basic local alignment search tools (BLASTn and BLASTx). The databases used for BLASTn and BLASTx were built from the viral reference sequences as described in Metavisitor pipeline [21]. An E-value cut-off of 0.01 was used. Following BLAST analysis, accession numbers were parsed to NCBI taxonomy files in R using package “taxonomizr” [24].

Taxonomic classification of identified viruses was done by conducting BLASTn and BLASTx on National Center For Biotechnology Information (NCBI) virus database and the obtained Blast XML files were imported into MEGAN6 program [25] and default parameters used. For comparison of viral content from the three study sites, assembled contigs recognized as viral after BLASTn and BLASTx were further classified into their respective taxonomic families or into a general group of unclassified viruses if not assigned a taxonomic family by the International Committee on Taxonomy of Viruses (ICTV).

### 2.5. Confirmation of Viral Sequences

Analyzed viral sequences were used to design specific PCR primers to confirm the presence of some selected viruses from the original RNA. PCR amplifications were carried out in a total volume of 25 μL consisting of 2 μL of template (cDNA), 0.25 μM of forward and reverse primers, 12.5 μL of PrimerSTAR Max Premix 2× (TaKaRa, Kusatsu, Japan), and Milli-Q ddH_2_O to supplement the system. Thermal cycling was initiated with a denaturation step at 94 °C for 5 min; followed by 35 cycles of 94 °C for 30 s, 58–60 °C 30 s and 72 °C for 1 min; and a final extension at 72 °C for 10 min. The PCR products were extracted with a Gel extraction kit (Omega Bio-Tek, Norcross, GA, USA) and sequenced at Tsingke Biological Technology. The primers used are presented in Table S1.

## 3. Results

### 3.1. Proportion of Viruses in Host Groups

In Kenya, we did not trap *Culex* spp. mosquitoes in sites within Kilifi County except sites in Kwale County where we dominantly trapped *Culex quinquefasciatus*. In China, we dominantly trapped *Culex tritaeniorhynchus* in all the sites within Hubei and Yunnan provinces. We sequenced 47 mosquito pools from Kwale, 100 pools from Hubei, and 100 pools from Yunnan. Mosquito samples from Kwale, Kenya generated a total of 21,747,508 raw reads. Samples from Hubei and Yunnan provinces, China had 6,714,707 and 36,277,174 raw reads, respectively. The average sequence reads lengths were 150 bp, 125 bp, and 398 bp for Kwale, Hubei and Yunnan respectively (Table 1).

**Figure 2:**
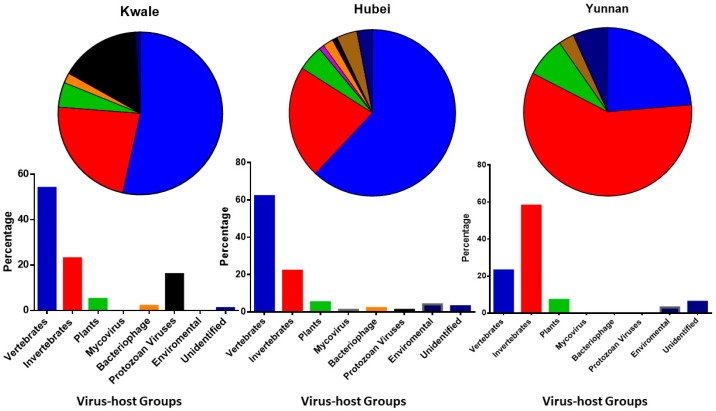
Percentage proportions of viruses in different host groups from mosquitos in Kwale, Kenya, and the Hubei and Yunnan provinces of China.

Our results showed that mosquitoes harbored viruses from diverse host groups: vertebrate viruses, invertebrate viruses, plant viruses, mycoviruses, phages, and protozoan viruses as well as groups that were classified as environmental and unidentified viruses (Figure 2). Across the three locations, abundant viruses (proportions > 5%) were those specific to host groups of vertebrates, invertebrates and plants, with exception of Kwale, which in addition had abundant protozoan viruses. Specifically, vertebrate host viruses were the most abundant in Kwale and Hubei. Invertebrate-specific viruses were the most abundant in mosquitoes of Yunnan Province (Figure 2).

### 3.2. Virus Families

**Figure 3:**
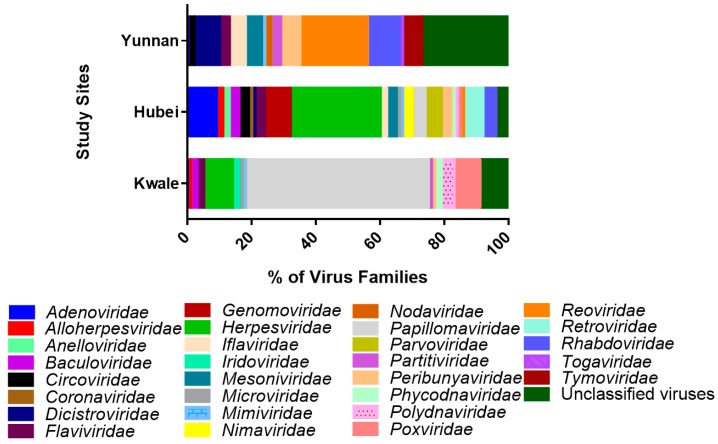
Stacked bar graph of all virus families (color coded) identified in the viromes of the *Culex* mosquito in Kwale county, Kenya and provinces of Hubei and Yunnan, China, 2014–2017. Only virus families with reads more than 30 were included in the figure.

Across the three locations, the mosquitoes harbored a total of 30 virus families and one general group of unclassified viruses (Figure 3). Vertebrate virus families include *Adenoviridae*, *Anelloviridae*, *Circoviridae*, *Coronaviridae*, *Flaviviridae*, *Herpesviridae*, *Papillomaviridae*, *Reoviridae*, *Retroviridae* and *Togaviridae*. Kwale had 14 virus families, with *Papillomaviridae* being the most abundant (Figure 3). Hubei had 18 virus families with *Herpesviridae* being the most abundant and Yunnan had 11 virus families, with the most abundant being unclassified viruses (Figure 3).

In Kwale, vertebrate, invertebrate, plant and protozoan viruses were the most abundant (Figure 2). The virome of Kwale mosquito was comprised of 14 virus families, with *Papillomaviridae* (57%) being most dominant, followed by *Herspesviridae* (8.17%). Among the invertebrate specific viruses, *Baculoviridae* was the notable virus family, whereas *Partitiviridae* (0.6%) was the leading plant virus. Kwale had a notable proportion of protozoan viruses, which were dominated by *Pandoravirus salinus* and *Pandoravirus dulcis* (unclassified viruses). These viruses were not identified in samples from China.

In the Hubei province, vertebrate, invertebrate, plant and unclassified viruses were the most abundant (Figure 2). *Herpesviridae* (30.16%) was the dominant vertebrate virus in the virome of Hubei followed by *Adenoviridae* (14%). *Nimaviridae* (3.6%) was the leading virus family in the invertebrate host group, while *Genomoviridae* (9.4%) was leading in part of plant viruses from Hubei.

**Figure 4:**
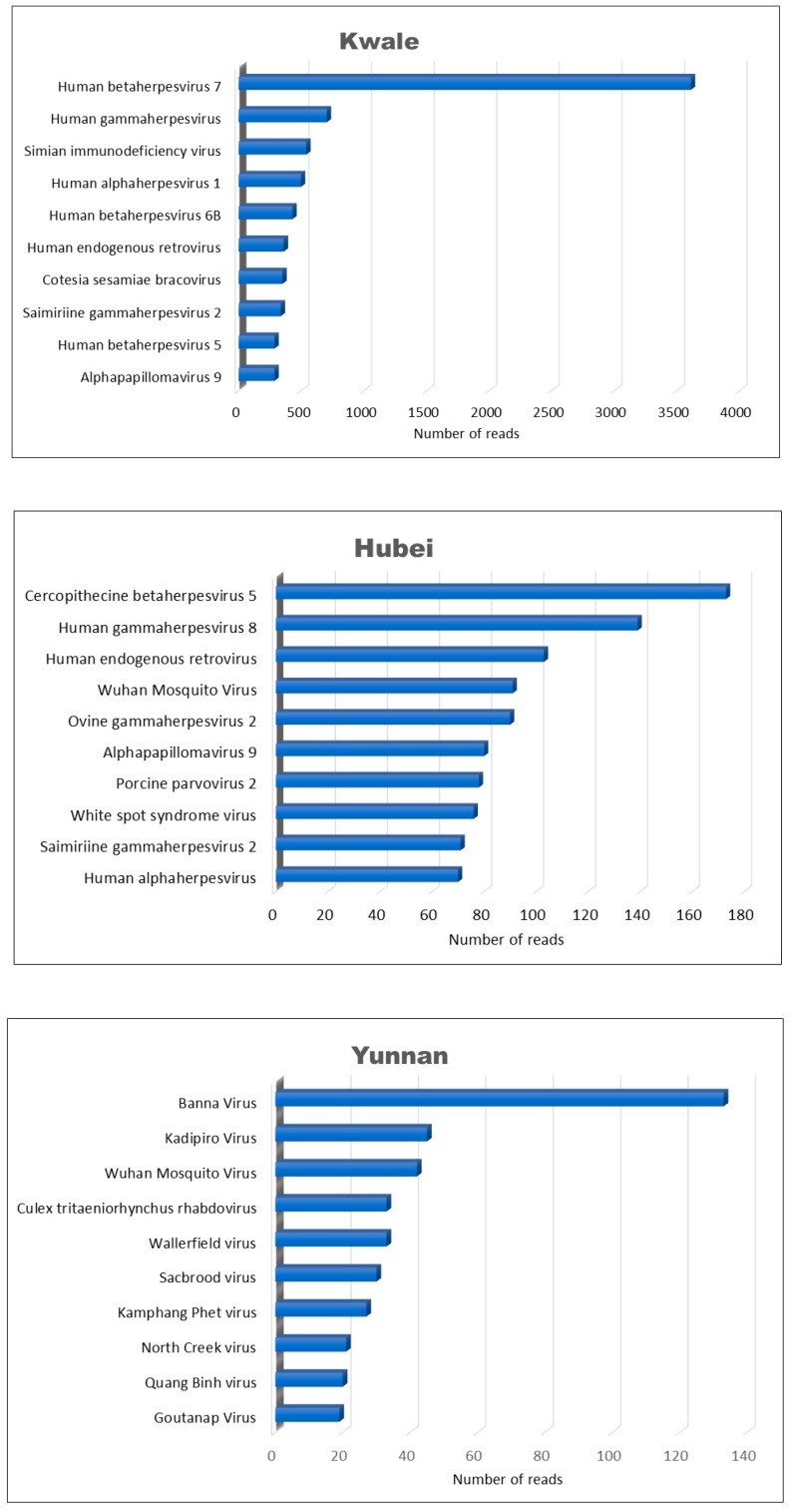
Abundance of the 10 most common virus species in *Culex* mosquito viromes in Kwale, Hubei, and Yunnan.

The virome of mosquito in the Yunnan province was dominantly composed of invertebrate-host related viruses (58.7%) (Figure 2) of which the majority were insect-specific viruses that included families *Iflaviridae*, *Dicistroviridae*, and *Mesoniviridae*, and some belonged to the unclassified virus groups. Our results showed that the Wuhan mosquito virus (unclassified viruses) and *Culex tritaeniorhynchus* rhabdovirus (*Rhabdoviridae*) were the most common virus species. In addition, Yunnan had a higher proportion of unclassified viruses (27.17%) compared to Kwale and Hubei. Under vertebrate viruses, the family *Reoviridae* (24.6%) was the most conspicuous group and it was dominated by the Banna virus (33%) and Kadipiro virus (11.6%) (Figure 4).

### 3.3. Viromes across the Three Locations

In the three study sites, the three most abundant virus families were *Flaviviridae* (Kwale—1.6%, Hubei—2.7% and Yunnan—2.5%) and *Herpesviridae* (Kwale—8.2%, Hubei—30%, and Yunnan—0.5%) as well as the group of unclassified viruses (Kwale—9.7%, Hubei—2.3%, and Yunnan—27.14%).

Under the family *Flaviviridae*, both Hubei (2.7% reads) and Kwale (1.6% reads) had flaviviruses that infected vertebrates and also some insect flaviviruses. Specifically, in Kwale we identified the dengue 2 virus (77 reads) which was further confirmed by PCR. *Culex* flavivirus (*Cx*FV) was the only insect-specific flavivirus identified in Kwale. Yunnan Province exclusively had insect-specific flaviviruses (2.5%), mainly dominated by the Quang Binh virus (QBV). In Hubei province, flavivirus was similarly dominated by QBV.

Additionally, the family *Mimiviridae* (protozoan-host) was present in all the three study sites, while *Totiviridae* (protozoan-host) was present in Yunnan and Kwale mosquitoes. Mycoviruses, bacteriophages, and environmental viruses contributed a significantly small percentage in all the three study sites.

## 4. Discussion

Emerging studies on mosquito viromes depict a highly diverse community of viruses whose existence, role, and transmission are less understood. Our results are thus consistent with previous studies as they revealed a rich diversity of both known pathogenic groups of viruses to humans and animals as well as viruses specific to other organisms (plants, protozoans, and invertebrates). Given that mosquito trappings in the three locations in Kenya and China were carried out at different times, our findings should be interpreted with that in mind. From our findings, Kwale, Kenya and Hubei, China (Figure 1) were comparable in virome profiles in that both were dominated by vertebrate viruses while virome profiles in Yunnan were dominated by invertebrate viruses, specifically insect-specific viruses (Figure 2). It thus appears that Kwale and Hubei had a high presence of vertebrates in the environment coupled by the probability that the sampled mosquitoes were mostly gravid and questing for blood meal. Interestingly, this was the first mosquito virome study in Kenya and provides a preliminary status.

Across the three locations, the mosquito viromes harbored a total of 30 virus families and one general group of unclassified viruses (Figure 3). The virus families included those specific to vertebrates, invertebrates, plants, protozoa, and bacteriophages. Since the three study sites in Kenya and China (Figure 1) were ecologically rich in mammalian and plant diversity (and were in close proximity to human settlements), the diversity was likely shaped by factors such as sex, whether female mosquitoes were gravid, the indiscriminate mosquito diet range (that includes plants and blood), and the micro-ecology supporting diversity of microorganisms, as well as contaminants in the environmental substrates they used for resting [26,27,28,29,30]. In the present study, we identified vertebrate viruses known to naturally infect mammals and replicate in mammalian cell lines, including *Adenoviridae, Anelloviridae, Circoviridae, Coronaviridae, Flaviviridae, Herpesviridae, Papillomaviridae, Reoviridae, Retroviridae, Togaviridae* and *Rhabdoviridae*. These virus families are of medical and veterinary importance and some have been identified as part of mosquito viromes elsewhere [3,31]. Across the three study sites, Hubei had the highest number of vertebrate-specific viruses, followed by Kwale, and the least were found in Yunnan (Figure 3), a variation that could be attributed to variation in the number of sampled blood-fed female mosquitoes. We did not quantify the sampled mosquitoes by sex, which we consider as one caveat of this study.

Among the vertebrate virus group in Yunnan (based on the top ten most abundant virus species), Banna virus and Kadipiro virus (family *Reoviridae*) had the most abundant viral reads in this set (Figure 4). These two are arboviruses of public health interest because they can infect vertebrates, including humans. Banna virus (BAV) and Kadipiro virus both contain double-stranded RNA with 12 segments and they were both classified under the genus Seadonavirus by the ICTV in 2016 [32]. Other known seadonaviruses are Liao ning virus [33], Balaton virus [34] and Mangshi virus [35]. In previous studies, BAV has been isolated within China from cattle, pigs, ticks, mosquitoes [36,37,38], and from the sera of febrile patients [39]. Although BAV has not been successfully propagated in mammalian cell lines, its seropositivity implies it can replicate in humans where it is speculated to cause encephalitis. BAV distribution has been shown to extend from tropical zones to the north temperate climate regions of Asia. Outside China, Banna virus has been isolated in Vietnam [40], Indonesia [41], and Inner Mongolia [42]. Wang and his team have also isolated BAV in Yunnan province, at China’s border with Laos and Myanmar and they suggest that BAV together with other lesser-known arboviruses might be silently circulating and infecting humans in the region [17]. Kadipiro virus was first isolated in 1981 from *Culex fuscocephalus* mosquitoes captured in Java, Indonesia. In China, the first isolation of Kadipiro virus was from *Culex tritaeniorhynchus*, *Anopheles sinensis*, and *Armigeres subalbatus* mosquitoes, all from the Yunnan province [43]. Similarly, as with BAV, propagation of Kadipiro virus in mammalian cell lines has not been reported yet.

Our results showed that viruses in the family *Herpesviridae* represent a significant component of the *Culex* mosquito virome, given that it was present in all the locations in China and Kenya. In addition, the virus family was most abundant in Hubei, coming second in Kwale, and was least prevalent in Yunnan (Figure 3). Herpesviruses are endogenous to a wide variety of vertebrate and invertebrate organisms and they infect a wide range of organisms, although through their ability to modulate host immunity, they are able to cause lifelong latent infections [44,45]. Our study identified several herpesviruses, with human betaherpesvirus 7 being dominant in Kwale while cercopithecine betaherpesvirus 5 was dominant in Hubei (Figure 4). Some of the human herpesviruses cause pathology in humans [46]. Out of the numerous herpesviruses latent in non-human primates only cercopithecine herpesvirus 1 is known to cause fatal illness in humans [47,48]. The presence and abundance of these herpesviruses suggest human and cercopithecine as easily available blood sources for mosquitoes in Hubei and Kwale. Further, mosquitoes are suggested to play a key role in the transmission of herpesviruses, such as the human herpesvirus-8 in humans [49,50,51]. Previous metagenome studies on mosquitoes [4,31], bats [52], and the most recent deep transcriptome sequencing of more than 220 invertebrate species [53] have all reported presence of herpesviruses from the three taxonomic subfamilies *Alphaherpesvirinae*, *Betaherpesvirinae* and *Gammaherpesvirinae* [45,54].

Further, our results showed that *Papillomaviridae* was a specifically dominant virus family in Kwale, while it was absent in Yunnan (Figure 3). Papillomaviruses are known to infect virtually all amniote vertebrates but they do not trigger a strong immune response [55,56]. In their natural hosts and their related species, papillomaviruses cause papillomas (benign tumors) in specific body sites such as the skin and mucosal epithelia. In both Kwale and Hubei, we identified alphapapillomavirus 9 (Figure 4), a species group associated with human cervical cancer which is responsible for 75% of all cases of cervical cancer worldwide [57]. However, overall, 80% of healthy human skin latently harbors diverse papillomaviruses [58]. It is therefore plausible that during blood meal from humans, the mosquito ingested these viruses.

Although not among the top ten virus species (Figure 4), but rather due to its public health importance, we detected dengue virus serotype 2 (flavivirus; *Flaviviridae*) in the Kwale mosquito virome. The virus causes variable symptoms ranging from mild febrile illness to the more severe dengue hemorrhagic fever, which can progress to a fatal form, dengue shock syndrome (DSS). *Aedes aegyptii* and *Aedes albopictus* are the two competent vectors of the virus worldwide [59,60,61]; hence, *Culex* mosquitoes ingested the virus from viremic hosts. The Kenyan coastal towns, which include Kwale, have experienced intermittent outbreaks due to dengue 2 in the past and presence of the virus suggests continuous low-level circulation.

It is worth noting that *Culex tritaeniorhynchus* is the main vector of the endemic Japanese encephalitis virus (JEV) and other zoonotic viruses like Banna virus and Kadipiro virus in Yunnan, Hubei and other provinces of China [17,62,63,64]. Although West Nile virus (WNV) is not endemic in China, it has been severally reported in the Xinjiang province [65,66,67]. In Kenya, there has been sporadic isolations of WNV in *Culex univittatus* from the Rift Valley [68], wild birds in the Tana River and Garissa Counties [69] and in ticks from north-eastern Kenya [70], We did not detect these important public health viruses from our mosquito viromes, which suggests that the sampled mosquitoes sourced blood meals from non-infected hosts. Perhaps a longer period of mosquito sampling is necessary to pick the virus especially if prevalence in the vertebrate hosts is low. Nevertheless, from our top ten vertebrate viruses, we inferred that the mosquitoes fed from a variety of vertebrate hosts. (Figure 4). Discovery of insect-specific viruses is on the rise, yet their role and impact on arbovirus transmission or potential pathogenicity is equivocal. However, they constitute a significant proportion in most of the mosquito viromes. Our study identified the virus families *Iflaviridae*, *Dicistroviridae* and *Mesoniviridae* as the most common, which is consistent with virome diversity and proportions of *Culex* spp. in Zambezi, Mozambique and Hubei, China [4,13]. Interestingly, previous studies showed that both *Culex* mosquito viromes in Zambezi and Hubei were dominated (up to 88%) by the insect-specific viruses, *Iflaviridae* [4,13]. In addition, we found that the Yunnan and Hubei mosquito viromes also had the Quang Binh virus (QBV), which is an invertebrate flavivirus. Still, Hubei was defined by the abundant *Nimaviridae*, that is usually composed of a single genus, *Whispovirus*, known to be of global occurrence and fatally infects crustaceans [71]. In contrast, the abundant insect-specific virus in Kwale virome was the *Baculoviridae*, a virus family that is known to infect diverse invertebrates but has also been reported to cause epizootics in field populations of *Culex* spp. [72]. Species from this family are being tested for use as mosquito control [73]. In addition, we identified *Culex* flavivirus (*Cx*FV), a virus that has previously been isolated across the world.

In the present study, we identified a notable proportion of plant-specific viruses across viromes in the three locations (Figure 2). We identified the family *Tymoviridae*—a group that constitutes globally distributed viruses—that causes significant losses in crops due to plant diseases [74]. Mosquitoes are not known to be vectors of plant-specific viruses even though due to feeding on plant sap and nectar, they ingest these viruses. Recently, a plant-specific virus, *Culex* originated Tymoviridae-like virus (CuTLV) (*Tymoviridae*), isolated from *Culex* spp. in China, showed cytopathic effect on mosquito cell lines, implying a potential for being vectored by mosquitoes [75].

In summary, mosquito virome studies are very few, implying that there is an enormous diversity of viruses yet to be uncovered. Our study was preliminary but it revealed that at a larger habitat scale, geolocation as well as the sampling time period are important factors that could enhance recovery of a greater diversity of mosquito viruses. We determined that each virome was unique in terms of virus proportions partly influenced by type of ingested meals (blood, nectar, plant sap, environmental substrates). Although vertebrate and other viruses ingested from other hosts are significant components of the virome, we suggest that since insect-specific viruses occur naturally in their hosts, they should be the basis for defining the mosquito virome. For instance, in this study, the dominant insect-specific viruses in Kwale, Hubei, and Yunnan were *Baculoviridae, Nimaviridae* and *Iflaviridae*, respectively, and hence such dominant viruses might have a role in the component community of the host. Our study is preliminary but due to emerging theories that suggest that manipulation of specific components of mosquito viromes could yield potential agents for control and prevention of pathogenic arboviruses and also the global emerging trends of mosquito-borne diseases, more studies are still necessary to uncover the global patterns of composition and diversity of mosquito viromes.

## Figures and Tables

**Table 1:** Summary of the sampling sites, mosquito species and sequenced data.

Country	Location	Coordinates	Mosquito Species	No. of Sequenced Reads	Average Length of Reads
Kenya	Kwale	4.46057° S, 39.47795° E	*Culex quinquefasciatus*	21,747,508	150 bp
China	Hubei	30.8843° N, 112.5923° E	*Culex tritaeniorhynchus*	6,714,707	125 bp
	Yunnan	24.9756° N, 101.4848° E	*Culex tritaeniorhynchus*	36,277,174	398 bp

## Supplementary Materials

The following are available online at www.mdpi.com/1999-4915/10/1/30/s1, Table S1: PCR primers used in validation of sequenced metagenomic data.
