# Supplementary material for: Metagenomic Virome Analysis of Culex Mosquitoes from Kenya and China
Source: Viruses. 2018 Jan 12;10(1):30. doi: 10.3390/v10010030 (PMC5795443; doi:10.3390/v10010030)
Supplement: Supplementary file 1 [file viruses-10-00030-s001.pdf]

Supplementary Table 1: PCR primers used in validation step of NGS sequences

	VIRUS	PRIMERS
1.	Banna Virus	FP: CTTCGCTAGGAGTCAAGGCT<br>RP: CTCTGGCTTGAATGTTTTCGC
2.	Circovirus 1	FP: CGCTAGCTCTTCGTTGTTCC<br>RP: GAGTCGGATTGCTGGGGAT
3.	Dengue	FP: CCAACACAAGGGGAACCCAG<br>RP: CATTGAAGTCGAGGCCCGTT
4.	Kadipiro Virus	FP: CCTGATCCTTCGGCACCA<br>RP: AGGGGATGGATCTTACCTGT
